# Safety and Effects of the Rapid Maxillary Expander on Temporomandibular Joint in Subjects Affected by Juvenile Idiopathic Arthritis: A Retrospective Study

**DOI:** 10.3390/children8010033

**Published:** 2021-01-07

**Authors:** Andrea Abate, Davide Cavagnetto, Francesca Maria Emilia Rusconi, Paolo Cressoni, Luca Esposito

**Affiliations:** 1 Department of Biomedical, Surgical and Dental Sciences, School of Dentistry, University of Milan, 20100 Milan, Italy; andreabate93@gmail.com (A.A.); davide.cavagnetto@gmail.com (D.C.); francesca.rusconi7@gmail.com (F.M.E.R.); paolo.cressoni@gmail.com (P.C.); 2 Fondazione IRCCS Cà Granda, Ospedale Maggiore Policlinico, 20100 Milan, Italy

**Keywords:** juvenile idiopathic arthritis, temporomandibular joint arthritis, maxillary hypoplasia, rapid maxillary expansion

## Abstract

Background: In Juvenile Idiopathic Arthritis (JIA) temporo-mandibular joints are often affected causing skeletal and dental malocclusions. The most frequent condition is mandibular hypoplasia, that may be associated with maxillary hypoplasia. The aim of this retrospective case control study is to investigate the effects and the safety of rapid maxillary expansion (RME) in growing patients affected by JIA. It was evaluated whether RME could be performed without complications on TMJs of JIA patients using DC/TMD protocol, and naso-maxillary transversal parameters were compared with the ones obtained on healthy patients. Methods: Twenty-five patients affected by JIA that ceased to manifest TMJ (Temporo-Mandibular Joint) symptoms in the previous year were treated with RME to solve the maxillary transverse hypoplasia. Postero-anterior cephalometric tracings were collected before and after treatment; linear measurements were obtained to study maxillary and nasal cavity modifications. Data were compared to those of a similar group of twenty-five healthy patients. Paired *t*-test and Independent *t*-test were used to evaluate changes before and after treatment in each group and to perform a comparison between the groups. Results: All patients demonstrated a statistically significant increase in nasal cavity width, maxillary width and upper and lower intermolar width. No patients presented a worsening of their TMJs condition. Intragroup comparisons revealed significant changes of cephalometric measurements, but no difference was found when comparing JIA and healthy patients. Conclusions: Growing patients with JIA that ceased to show signs of active TMJ involvement for at least one year could be safely treated with RME, expecting similar benefits to those of healthy patients. Dentists and rheumatologists should be informed of safety and potential benefits of palatal expansion in JIA patients in order to improve the outcome of orthodontic treatment and reduce the indication for more invasive procedures (i.e., Surgical Assisted Rapid Maxillary Expansion).

## 1. Introduction

Juvenile Idiopathic Arthritis (JIA) is a heterogeneous group of conditions of unknown aetiology that affects the connective tissue of joints for at least six weeks, before the 16th year of age [1]. Since reported incidence and prevalence in the population range from 2 to 20 and from 16 to 150 per 100,000 respectively, JIA is considered to be one of the most common rheumatic diseases in childhood in both Europe and North America [1].

In 38% to 72% of cases, the temporomandibular joint (TMJ) is involved among other anatomical regions, depending on the diagnostic method used and the JIA subtype examined [2,3,4,5]. The TMJ can be affected unilaterally or bilaterally, with pain-impaired functional disorders, impaired bite force and tenderness of masticatory muscles resulting in severe disabilities [6,7,8,9,10]. TMJ involvement may produce relevant sagittal and vertical growth disturbances in both jaw resulting in severe micrognathia, anterior open bite, marked asymmetry and posterior cross-bite [11,12,13,14]. The optimal approach for JIA treatment is based on a multidisciplinary approach with nonpharmacological and pharmacological interventions [1,15]. Orthodontic and gnathological treatment is of primary interest to investigate TMJ involvement and to guide sagittal and vertical mandibular growth until a harmonic development of both jaw is accomplished [3,16].

Few studies describe orthodontic treatment protocols for JIA patients [3,16,17,18,19,20]. Functional appliances such as Andresen activators or distraction splints are used in these patients to promote mandibular growth, to correct mandibular asymmetry and/or to reduce intermaxillary divergence. There is no concordance about interceptive treatment effectiveness because JIA patient’s response to stimulation through functional treatment is reduced compared to controls due to the rheumatic condition and differs between patients [21]. On the other hand, the self-awareness of the condition brings a better compliance to the treatment [22].

Recent literature reviews stated the importance of orthodontic treatment in growing JIA patients, but no guidelines were proposed about treatment protocols to follow [16,18,23].

As far as the authors are aware of available literature on orthopaedic treatment in JIA patients is scarce [5,23].

In 2017 Menezes et al. described a case treated with a quad-helix appliance, followed by a Nance lingual arch in mandible with six years of follow up. However, they did not analyze the effect on the maxilla other than crossbite correction [24] Juvenile idiopathic arthritis is still variably associated with maxillary hypoplasia and, in particular with posterior cross bite in the primary dentition [25]. Growing patients suffering from JIA with TMJ involvement may present maxillary hypoplasia and would benefit from maxillary expansion as has been proven in healthy patients [26]. The rapid maxillary expander (RME) is recognized as one of the best treatment options for unilateral/bilateral cross bite and severe maxillary crowding in growing patients with maxillary hypoplasia [27]. However, no studies described the use of this appliance in patients affected by JIA. Atraumatic orthopaedic appliances like distraction splints and Andresen activators seems to be preferred in case reports and case-series [23]. Drawbacks of the expansions obtained with Andresen or Quad-helix appliances are the biological limitations of maxillary dentoalveolar expansion and a prolonged phase when the slow transversal movement of posterior lateral teeth can develop premature contacts during functional movements (i.e., chewing) that are harmful to TMJ health [28,29,30]. RME is indeed a fast option to solve cross bite and premature contacts within 10 to 15 days. Even though it is perceived as more traumatic because of its rapidity of action it has been proven to cause no harm to temporo-mandibular joints [31,32,33]. The benefits of RME include augmentation of condylar space and the mandible repositioning forward thus reducing condylar functional stress [33,34] and improving skeletal second class [35,36].

Other positive effects of RME that have already been proven in healthy patients include promotion of a statistically significant increase in SNB angle [37], improvement in nasal respiration and in symmetry of facial structures [38,39]. These adjunctive effects would be also helpful in the treatment of JIA patients as their most common facial characteristic is mandibular hypoplasia and facial asymmetry [11,12].

The aim of this retrospective study was to analyze whether RME could be performed without complications on TMJs of JIA patients using DC/TMD protocol, and to compare naso-maxillary transversal parameters with the ones obtained on patients without JIA.

## 2. Materials and Methods

### 2.1. Study Design, Type of Participants and Inclusion Criteria

A retrospective study was performed analyzing postero-anterior cephalograms prior and following the expansion of patients affected by JIA and comparing them with healthy controls.

The study protocol was approved by the competent Institutional Review Board (IRB) as part of the research project of the year 2018 O.U. N. 420/425 of Fondazione IRCCS Cà Granda Ospedale Maggiore Policlinico, Milano. All patients’ parents provided written informed consent to all the procedures performed.

Twenty-five records of Caucasian patients (8 male and 17 female, mean age 9.4 ± 1.2) with a diagnosis of JIA with TMJ involvement treated with rapid maxillary expansion for maxillary hypoplasia (posterior unilateral or bilateral cross-bite or severe crowding with no cross-bite) were selected from the database of the Department of Biomedical Surgical and Dental Sciences, University of Milan, Italy.

Twenty-five records of Caucasian healthy patients (10 men and 15 women, mean age 8.6 ± 1.8) treated with RME with the same diagnosis served as controls.

Each JIA patient was assessed according to the International League of Associations for Rheumatology (ILAR) criteria (15 cases oligoarticular JIA, 10 polyarticular JIA), with a mean age of 6.8 years at disease onset [40,41]. The severity of TMJ inflammation seen on Magnetic Resonance Imaging (MRI), that was taken as control before expansion, was assessed based on the grading system developed by Cahill [42]. All patients were in Age Grade 3 (presence of juxta-articular erosions) with no sign of an active inflammatory process. TMJ involvement at baseline was assessed as negative according to DC-TMD IIIA [43]. All of them were in a quiescent phase of the disease. They used to take methotrexate during the active phase of the pathology and started to be treated at the Department of Orthodontics, twelve months after their last dose of medication [5].

Inclusion criteria for the JIA group were: diagnosis of JIA with unilateral TMJ involvement, treatment started during a JIA quiescent phase that lasted for at least 12 months, transverse maxillary hypoplasia treated with a rapid maxillary expansion protocol with a hyrax type expander, presence of postero-anterior radiographs before and after expansion (mean distance between radiographs: 6 ± 3 months in JIA group and 7 ± 4 months in control group); records reporting the assessment of TMJ involvement according to DC-TMD IIIA at baseline, at the end of maxillary expansion, and again after 1 month, 3 months and 6 months during follow-up appointments; no previous orthodontic treatment.

Inclusion criteria for the control group were transverse constriction of the maxilla with unilateral or bilateral cross bite; presence of postero-anterior radiographs before and after expansion; no previous orthodontic treatment. The exclusion criteria in both groups were congenital anomalies, dental anomalies (number, form and dimension of teeth), previous orthodontic treatment.

All patients from both groups were treated between 2017 and 2018 in the Orthodontic Department of Fondazione IRCCS Cà Granda Ospedale Maggiore Policlinico of Milan (University of Milan) with Hyrax palatal expander to correct maxillary transverse hypoplasia, to treat posterior cross bite and to increase the space within the arch.

**Figure 1:**
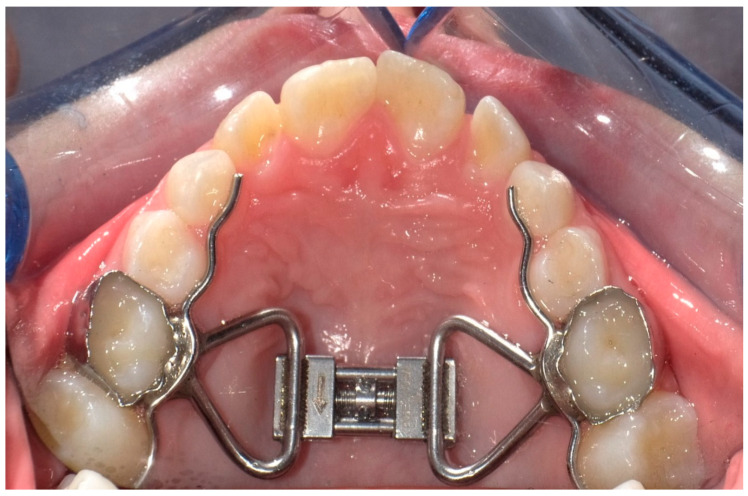
Rapid Palatal expander banded on the second deciduous molars.

All patients were treated with a Hyrax-type rapid maxillary expander bonded to maxillary second deciduous molars with a glass ionomer cement (Multi-Cure; Unitek, Monrovia, CA, USA) (Figure 1). Four activations were performed chairside, two activations by the orthodontist and two by the patient’s parents as training. Activations were prescribed twice a day (0.50 mm) for 7 days until the next follow-up appointment when patients would be re-evaluated, and the clinician would decide whether to stop or continue the activations. The screw was activated until dental overcorrection, that is when palatal cusps of the upper first permanent molars occluded onto lingual side of buccal cuspids of the lower first permanent molars [44]. After the active expansion phase, the screw was locked with light-cure flow composite resin (Transbond Plus Band adhesive; Unitek, Monrovia, CA, USA). The active treatment lasted between 15 and 21 days. The appliance was kept in place for 6 months for retention.

### 2.2. Cephalometric Analysis

Digital teleradiograps in postero-anterior projection of all patients were taken with the same machine (Sirona^®^ Orthophos XG) with fixed focus sensor distance (150 cm) at the Diagnostic Imaging Unit of the Fondazione IRCCS Cà Granda, Ospedale Maggiore Policlinico, Milan, Italy. Cephalometric tracings were performed by an operator using a cephalometric analysis software (Deltadent-Piolla, Milan, Italy) that computed all reported indexes. One month later the tracings were made by a different operator and traced again by the first one in order to evaluate intra- and interoperator variability on 20 randomly selected postero-anterior radiographs. Intraclass correlation coefficients (ICC) were calculated to compare within-subject variability to between-subject variability; correlation coefficients results were larger than 0.93. The method error was considered negligible.

**Figure 2:**
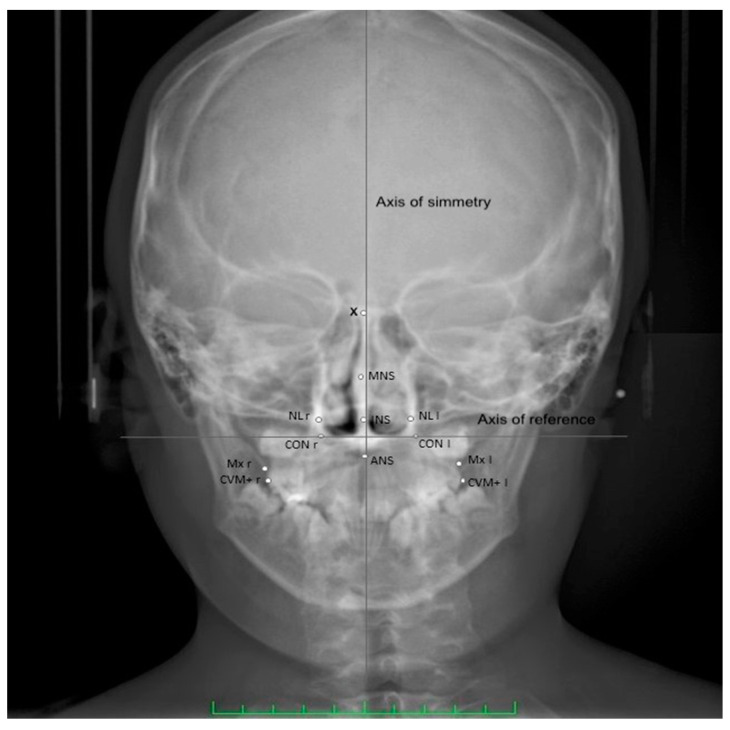
Cephalometric landmarks used in the present study. Definitions of the landmarks are presented in Table 1.

Twelve points from posteroanterior cephalometry were considered in the study to evaluate maxillary transverse dimension and nasal cavity width (Table 1) (Figure 2) [38]. The height discrepancy between homologous points to the axis of symmetry was also considered. The width of the nasal cavity and the maxillary width (Mx l-r) and upper intermolar width (CVM+ l-r) have been measured. The linear difference between the following homologous points to the axis of symmetry has been also considered CVM+ l-r as well as the distance of the upper and lower midline points to the axis of symmetry.

### 2.3. Statistical Analysis

Sample size was calculated a priori to obtain a statistical power of the study greater than 0.95 at an alpha of 0.05, using the mean values and standard deviations of maxillary width assessed with postero-anterior cephalometric study by Lanteri et al. [45]. The values of the mean difference maxillary width before and after treatment was used to perform the power analysis calculation along with the corresponding standard deviations. The data used to perform the analysis were: mean difference Maxillary width = 4.2; σ = 3.6; α = 0.05; δ = 95 β = 0.05. Based on these parameters, to have an 95% chance of detecting as significant (at the two-sided 5% level) maxillary width difference between the two time points the sample size required was 10 patients. Statistical analyses were conducted using SPSS ^®^ 23 for Windows (IBM, Sommers, NY, USA). The Shapiro–Wilk test was used to assess whether the data were normally distributed. The statistical distribution of the quantitative measures was found to be Gaussian. Each measurement has been reported as mean and standard deviation. Paired *t*-test was applied to evaluate any difference between all the variables evaluated (Mx r-l; Cvm+ r-l; NL r-l; MNS-MID; INS-MID; X-MNS; MNS-ANS; CVM+ r-l- axis) in both JIA and the control group (measurements carried out on the same patients before and after treatment).

Independent *t*-test was used to evaluate whether the difference before and after treatment of each measurement (Mx r-l; Cvm+ r-l; NL r-l; MNS-MID; INS-MID; X-MNS; MNS-ANS; CVM+ r-l- axis) between the two groups was significantly different. A *p*-value < 0.05 was considered statistically significant.

## 3. Results

None of the patients, neither in the JIA nor in the control group, reported experiencing any spontaneous pain at the level of either joint during lateral excursion, protrusive excursion, unassisted maximum opening or function during all follow-up visits (immediately after expansion, 1 month, 3 months and six months after). Thus, all patients responded negative according to DC-TMD IIIa criteria (Table 2) [43].

Descriptive statistics and statistical comparisons of maxillary linear (mm) measurements before (T0) and after treatment (T1) with RME are shown in Table 3 and Table 4. Both in the JIA group and in the control group all measurements before and after treatment showed statistically significant differences (*p* < 0.05).

### 3.1. Linear Measurements

The average mean (SD) maxillary width (MX r-l) increase was 3.07 (2.19) mm in JIA group and of 2.94 (1.84) in the control group, the average upper intermolar width (Cvm+ r-l) increased 6.08 (3.77) mm in JIA group and of 5.67 (2.86) mm in the controls. The width of nasal cavity increased in average 2.92 (2.32) mm in JIA group and 3.29 (2.21) mm in control group.

### 3.2. Symmetry Indexes

The height discrepancy to the axis of symmetry between CVM+ right and left improved −1.07(0.21) in JIA group and −1.26(0.15).

Results obtained from the two groups were compared with Independent *t*-test for normally distributed data and no statistically significant differences were found between them for all the measurements (Table 5).

## 4. Discussion

Few articles deal with orthodontic treatment in patients suffering from JIA [17,46]. Much of the evidence derives from case reports and case series [24,37]. Only two studies evaluate larger samples [17,46]. There is little evidence regarding orthodontic treatment on JIA patients that would allow us to draw a clear evidence based conclusion [3,16].

Resnick et al. in a report in 2019 during a multinational consensus conference stated that in a skeletally immature patient with no or minimal active TMJ disease a functional orthopedic treatment is recommended until skeletal maturity; this may avoid the need for a surgical orthodontic management [47].

Since palatal constriction could be observed in this type of condition [48], we decided to evaluate the effects of the maxillary rapid expander as it has not yet been described in the literature to assess its effects and possible contraindications in JIA patients in the quiescent stage of the disease. The early use of tooth-born RME avoids surgical or more invasive operations when maturation is completed without an exacerbation of the disease [49]. The maxillary expansion for solving cross bite and crowding has several positive effects, avoiding the worsening of the asymmetry and eliminating dental interferences that could cause a worsening of the joint symptomatology and subsequently in mandibular asymmetry [28,29,30,31,32,33,50,51]. Premature contacts in the primary canine region, for example, is the most prevalent cause of functional mandibular shift [52] and can lead to TMJ problems since the prevalence of self-correction of such malocclusion is very low. Several studies have revealed that it is possible to promote anterior positioning of the mandible when the maxilla is expanded transversely in subjects with Class II malocclusions [26,37,53]. Palatal expansion also releases the mandible to move forward, thus promoting the mandible to grow [54], helping in Class II correction which is the most common maxillofacial characteristics of JIA patients [11,12,22].

In our study, a statistically relevant enlargement in the transverse dimension of the palate was found after RME in JIA and control patients, both groups demonstrated an increase of the maxillary width (Mx l-r) and of the upper intermolar width (CVM+ l-r). Moreover, all results obtained from both groups are comparable to previous studies that described the effect of rapid maxillary expansion appliances in the literature [55]. RME proved to be effective also on the nasal cavity, increasing volume and width of nasal airways, and reducing septal deviation both in its middle and lower tracts.

Our results showed relevant improvement in all measurements both in test and control group, since the height discrepancy between points decreased and the nasal cavity width increased. No relevant difference was found between the test and the control group in concordance with Authors’ expectations. The response to treatment is not affected by this systemic condition although the benefits of RME treatment could have a strong positive impact on skeletal malocclusion and TMJ health of JIA patients. In particular the augmentation of condylar space and the mandible repositioning forward thus reducing condylar functional stress [33] and improving skeletal second class [35]. Literature has already described positive effects on systemic conditions after the enlargement of upper airways by RME, such as the improvement in the respiratory nasal pattern that allows a greater gas exchange at the alveoli in the lungs [56]. According to our results, we can expect JIA patients can enjoy these systemic benefits, too [57].

Our patients treated with RME during a nonactive phase of pathology did not develop TMJ complications in the TMJs after expansion and in the following months. Palatal expansion seems to be therefore a safe procedure in JIA patients. A study on the effect of RME on mandibular growth in JIA patients in the future is essential to be able to evaluate the real benefits of palatal expansion on mandibular growth of these patients.

Certain limitations of the study should be noted including: the analysis focused only on the upper jaw, not considering the mandible and the use of a two-dimensional cephalometric analysis. However, the aim of this study was fulfilled as JIA patients were successfully treated with RME without any TMJ complications. Future studies could focus on quantifying sagittal effects on lateral cephalograms connected to mandible repositioning after maxillary expansion. The high significance achieved for all measurements allows us to clearly conclude that JIA patients undergoing RME have experienced benefits similar to those of healthy patients without any complications.

The number of patients included in this study, although sufficient to identify statistically significant differences, is small to generalize its findings. Future studies should investigate a larger sample of subjects affected by JIA considering also the subtype of JIA present. It would also be of a certain interest to monitor in a prospective study TMJ inflammation through MRI examinations taken immediately after rapid maxillary expansion and at a six month follow-up and compare them to baseline MRI in order to completely exclude any detrimental effect on TMJs, although it is occasionally difficult to perform MRI in paediatric patients [58].

## 5. Conclusions

Orthodontists in cooperation with pediatric rheumatologists should be trained to recognize dentofacial alterations in patients suffering of JIA. Regular clinical examination and early treatment, together with progressive monitoring of the craniofacial development is advised. Treatment of JIA patients in the quiescent phase with maxillary constriction using RME is a suitable option for all patients that show no signs of disease relapse at TMJ level for about 1 year. Clinical indications are the same as for healthy patients. In both, it was possible to observe an improvement in nasal and maxillary asymmetry indexes. From a clinical point of view the predetermined therapeutic goal was reached. There was a resolution of the mono/bilateral cross bites and/or of dental crowding, an increase in transverse dimension of the maxilla that could promote anterior positioning of the mandible and help in Class II correction. All these positive effects can be accomplished without any manifestation of joint damage. Further studies on RME in patients affected by JIA should be performed to thoroughly examine its effects on these patients’ craniofacial conditions.

## Figures and Tables

**Table 1:** Landmarks used and their definition.

Abbreviation	Definition
CVM+ l/r	Most prominent point on the sagittal plane of the vestibular-mesial cuspid-upper left and upper right first permanent molar (left and right)
MNS	Middle Nasal Septum-middle point of the maximal diameter of the medium third of nasal septum, on the horizontal plane
INS	Inferior part of the Nasal Septum-middle point of the maximal diameter of the inferior third of nasal septum, on the horizontal plane
MID	Crossing point between the axis of symmetry and the horizontal straight line that connects the homolog cephalometric points
CON	Point of cephalometric congruence between the inferior condyle of the occipital bone and the contour of the great occipital foramen
Axis of reference	Axis passing through right and left CON
Axis of symmetry	Perpendicular to the reference axis, passing through the highest point of the occipital foramen
NL	External points at the maximal horizontal diameter of nasal cavity, left and right
X	Crossing point between the perpendicular plate of the ethmoid and the projection of the floor of the anterior cranial fossa floor
ANS	Anterior cephalometric nasal spine
Mx l/r	The intersection of the lateral contour of the maxillary alveolar process and the lower contour of the maxillozygomatic process of the maxilla (left and right)

**Table 2:** Summary of the general characteristics of Juvenile Idiopathic Arthritis (JIA) patients analyzed.

Characteristics of JIA Group	
Sex	10 men and 15 women
Age	Mean age 8.6 ± 1.8
Follow up time	6 ± 3 months
JIA Subtypes	15 cases oligoarticular JIA, 10 polyarticular JIA
Severity of TMJ inflammation	All patients were in Age Grade 3 (Cahill grading system)
Disease onset	Mean age of 6.8 ± 1.1 years
TMJ involvement at baseline	Negative according to DC-TMD IIIA. All of them were in a quiescent phase of the disease

**Table 3:** JIA patients. Mean, standard deviation (SD) and comparisons of pre-treatment (T1) and post-treatment (T2) values within the Group RME and comparison of the measurements with paired *t*-test. NS = not significant.

Measurements	TO (*n* = 25) Mean (SD)	T1 (*n* = 25) Mean (SD)	Δ T1-T0	Significance
Mx r-l	59.24 ± 3.03	62.31 ± 2.55	3.07	0.0023
Cvm+ r-l	56.31 ± 2.73	62.40 ± 2.88	6.08	0.0089
NL r-l	26.79 ± 3.41	29.71 ± 3.22	2.92	0.0095
MNS-MID	1.33 ± 0.26	0.14 ± 0.22	−1.19	0.0074
INS-MID	0.88 ± 0.34	0.03 ± 0.18	−0.85	0.0262
X-MNS	23.81 ± 0.91	25.17 ± 0.83	1.36	0.0464
MNS-ANS	23.91 ± 0.32	25.19 ± 0.24	1.28	0.0398
CVM+ r-l- axis	2.43 ± 0.20	1.16 ± 0.20	−1.07	0.0413

*p* < 0.05 was considered as statistically significant.

**Table 4:** Control patients. Mean, standard deviation (S.D.) and comparisons of pre-treatment (T1) and post-treatment (T2) values within the Group RME and comparison of the measurements with paired *t*-test. NS = not significant.

Measurements	TO (*n* = 25) Mean (SD)	T1 (*n* = 25) Mean (SD)	Δ T1-T0	Significance
Mx r-l	58.53 ± 2.92	61.47 ± 2.07	2.94	0.0092
Cvm+ r-l	56.31 ± 0.68	61.98 ± 0.78	5.67	0.0036
NL r-l	27.74 ± 2.91	31.03 ± 2.88	3.29	0.0071
MNS-MID	1.01 ± 0.2	0.15 ± 0.1	−0.86	0.0264
INS-MID	1.01 ± 0.13	0.07 ± 0.09	−0.94	0.0359
X-MNS	23.91 ± 1.14	24.97 ± 0.93	1.16	0.0178
MNS-ANS	23.93 ± 0.51	24.98 ± 0.67	1.05	0.0472
CVM+ r-l- axis	2.47 ± 0.17	1.21 ± 0.18	−1.26	0.0403

*p* < 0.05 was considered as statistically significant.

**Table 5:** Mean, standard deviation (S.D.) of measurements between T0-T1 and comparisons of values within JIA group and Control group with independent *t*-test. NS = not significant.

Measurements	JIA Δ T1-T0 (*n* = 25) Mean (SD)	Control Δ T1-T0 (*n* = 25) Mean (SD)	Δ JIA-Control	Effect Size (Cohen’s d)	Significance
Mx r-l	3.07 ± 2.19	2.94 ± 1.84	0.13	0.092	0.835
Cvm+ r-l	6.08 ± 3.77	5.67 ± 2.86	0.41	0.23	0.181
NL r-l	2.92 ± 2.32	3.29 ± 2.21	−0.37	−0.25	0.594
MNS-MID	−1.19 ± 0.11	−0.86 ± 0.17	−0.33	−0.88	0.121
INS-MID	−0.85 ± 0.12	−0.94 ± 0.08	0.09	0.28	0.753
X-MNS	1.36 ± 0.87	1.16 ± 0.79	0.21	0.22	0.237
MNS-ANS	1.28 ± 0.71	1.05 ± 0.56	0.23	0.29	0.296
CVM+ r-l- axis	−1.07 ± 0.21	−1.26 ± 0.15	0.19	0.45	0.465

*p* < 0.05 was considered as statistically significant.

## Data Availability

The data presented in this study are available on request from the corresponding author.
